# Therapeutics of Cholera

**Published:** 1893-12

**Authors:** 


THERAPEUTICS OF CHOLERA (CHOLERA ASIATICA). By P. C. Majumdár, M. D. Philadelphia: Bœricke & Tafel. 1893. Price, 50c., net.

This little book of one hundred duodecimo pages is the latest contribution to the literature of cholera. It constituted an essay originally read before the World's Homœopathic Congress at Chicago, in connection with the World's Columbian Exposition, 1893. It is an excellent work, is strictly homœopathic, and is furnished with a materia medica and repertory. It is additionally interesting as being the production of a native Hindu.

